# Severe Myocardial Involvement and Persistent Supraventricular Arrhythmia in a Premature Infant Due to Enterovirus Infection: Case Report and Literature Review

**DOI:** 10.3390/jcdd12060228

**Published:** 2025-06-14

**Authors:** Carolina Montobbio, Alessio Conte, Andrea Calandrino, Alessia Pepe, Francesco Vinci, Alessandra Siboldi, Roberto Formigari, Luca Antonio Ramenghi

**Affiliations:** 1 Neonatal Intensive Care Unit, Department of Maternal and Neonatal Health, IRCCS Istituto Giannina Gaslini, 16147 Genoa, Italy; carolinamontobbio@gaslini.org (C.M.); andrea.calandrino@edu.unige.it (A.C.); francescovinci@gaslini.org (F.V.); lucaramenghi@gaslini.org (L.A.R.); 2 Department of Neuroscience, Rehabilitation, Ophthalmology, Genetics, Mother and Child Health, School of Medical and Pharmaceuticals, University of Genoa, 16132 Genoa, Italy; 5121887@studenti.unige.it; 3 Pediatric Cardiology Department, IRCCS Istituto Giannina Gaslini, 16147 Genoa, Italy; alessandrasiboldi@gaslini.org (A.S.); robertoformigari@gaslini.org (R.F.)

**Keywords:** enterovirus, myocarditis, preterm, ELBW, supraventricular tachycardia, arrhythmia

## Abstract

Enterovirus (EV) infections in neonates can be transmitted vertically or horizontally, with symptoms ranging from mild to severe, including myocarditis, meningoencephalitis, and hepatitis. Neonates with EV-induced myocarditis may present severe cardiovascular disease with sudden onset of arrhythmia. Neonatal arrhythmias, particularly in low birth weight or critically ill infants, can impair cardiac function and worsen outcomes. EV targets cardiomyocyte receptors, inducing apoptosis pathways and triggering cardiac conduction disturbances. We present an extremely low-birth-weight preterm infant (GW 27 + 6) who developed EV-induced myocarditis, complicated with a sudden onset of supraventricular tachycardia (SVT), pericardial effusion and bi-atrial enlargement. Despite multi-agent regimen, including propranolol, flecainide, and amiodarone, the infant showed persistent junctional rhythm until seven months of age, later transitioning to atrial rhythm with stable cardiac function. A review of previously published rhythm disturbances due to EV-induced myocarditis is presented. Newborns with EV-induced arrhythmia may require a multi-modal treatment such as a multi-agent medical regimen or, in severe non-responsive cases, an electrophysiological approach. EV infections may cause long-term cardiovascular comorbidities (such as left ventricular dysfunction or mitral valve regurgitation), necessitating continuous monitoring through echocardiography and ECG. Collaboration between neonatologists and pediatric cardiologists is crucial for effective treatment and follow-up.

## 1. Introduction

Enterovirus represents a common cause of infection in the neonatal intensive care units (NICU) [1]. The route of transmission can be vertical, occurring before, during, and after delivery, or horizontal, involving contact with family members or healthcare professionals [2]. Seasonal variation or clustering in enterovirus infection has been described [3].

Infection can be mildly symptomatic (up to 80% of patients [4]) or severe, with the development of myocarditis, meningoencephalitis, hepatitis and other life-threatening conditions [2]. Maternal flu-like symptoms or diarrhea in the period from 2 months prepartum to 7 days postpartum have been reported in 30% of severe enterovirus infections [2]. However, the presence of symptoms at least 5–7 days before delivery is associated with a milder course in the neonate (due to specific maternal antibodies crossing the placenta) [3]. In about 70% of cases, the onset age of symptoms is less than 7 days of life [2].

Classic clinical symptomatology is characterized by fever or hypothermia, rash, lethargy, hyporexia, or respiratory distress. In cases of severe illness, the presence of heart failure with shock, arrhythmias, or seizures may be present [2].

In a systematic review regarding severe enterovirus infection, 37.1% of patients had myocarditis, of which approximately one-third developed arrhythmias (30.7%). Abnormalities observed in electrocardiograms may include sinus tachycardia, T wave abnormalities, ventricular tachycardia (VT), and supraventricular tachycardia (SVT) [1]. ECG signs of inflammatory involvement may be present in up to 30% of cases [2]. The prognosis for neonates who develop myocarditis associated with enterovirus infection is variable, ranging from a self-remitted course to the necessity for extracorporeal membrane oxygenation (ECMO). Overall mortality for enterovirus-related myocarditis is estimated to be 38%, reaching 67% in patients requiring ECMO [4].

The prevalence of neonatal arrhythmias (NAs) is reported to be between 1% and 5% in NICU [5]. NAs can be classified as benign or non-benign, defined as the capacity to result in hemodynamic compromise or clinical deterioration. Regarding neonatal patients, non-benign arrhythmias (iterative SVT, AV dissociation, VT, ventricular fibrillation (VF), and long QT syndromes) have the highest prevalence in extremely low-birth-weight individuals (ELBW), while benign NAs are more common in low-birth-weight (LBW) and moderate-to-late preterm neonates [5]. As demonstrated by the Frank–Starling law, the limited functional reserve of the newborn heart means that any significant change in heart rate will result in a decline in cardiac output, impaired cardiac filling and venous congestion [6]. This effect is more significant for premature neonates, those critically ill, or those with concomitant congenital heart disease [5].

SVT is one of the most common arrhythmic emergencies in newborns, occurring in approximately 1 in 250 to 1 in 1000 live births. SVT in neonates and infants arises chiefly from reentrant circuits [7]. Atrioventricular reentrant tachycardia (AVRT) via an accessory pathway (e.g., Wolff–Parkinson–White) is the predominant mechanism, accounting for 70–80% of cases in infancy. Atrioventricular nodal reentrant tachycardia (AVNRT), due to dual slow and fast AV nodal pathways, occurs in 5–17% of patients. A rarer but clinically important variant, permanent junctional reciprocating tachycardia (PJRT), employs a slowly conducting posteroseptal accessory pathway to sustain incessant reentry and predispose to tachycardia-induced cardiomyopathy [8]. Far less common substrates include Mahaim fiber-mediated antidromic circuits and junctional ectopic tachycardia (JET) driven by His bundle automaticity. Although most neonatal supraventricular tachycardias are due to congenital accessory conduction pathways, some of these arrhythmias may be acquired, for example, as a result of acute inflammatory injury in cases of myocardial involvement. Both viral and bacterial pathogens may cause such potentially dramatic complications [9,10].

To our knowledge, this is the first reported case of supraventricular tachycardia, secondary to enteroviral myocarditis in an extremely low-birth-weight (ELBW) preterm infant with fetal growth restriction. This case provides unique insight into the diagnostic and therapeutic challenges faced in such a vulnerable population, particularly in the context of persistent arrhythmia and myocardial involvement requiring prolonged multi-drug treatment and long-term monitoring.

## 2. Case Report

A 27 + 6 GW (gestational weeks) female infant was born by urgent C-section due to preeclampsia, HELLP syndrome, and pathological Doppler sonography of the placental vessels. Prenatal ultrasound showed fetal growth restriction. Steroid prophylaxis with betamethasone was completed before delivery. No history of fetal arrhythmias had been reported. The birth weight was 760 g and APGAR score was 3, 5, and 7 at 1, 5, and 10 min, respectively. Umbilical arterial blood gas was normal (pH 7.19). Due to worsening respiratory distress (Silverman 4–5) and oxygen requirements (FiO_2_ up to 100%), the patient was intubated in the delivery room and transferred to NICU. During positive pressure ventilation, endotracheal surfactant therapy (Curosurf 200 mg/kg) was administered on the first day of life with improvement in clinical condition. Antibiotic empiric treatment was started (Ampicillin 100 mg/kg BID and Gentamicin 5 mg/kg/die) and then suspended in the absence of signs of early onset sepsis.

On the third day of life, due to the persistence of mild respiratory distress, a second administration of surfactant was given (Curosurf 100 mg/kg). Simultaneously, echocardiography showed a hemodynamically significative patent ductus arteriosus (PDA) with a left-to-right shunt. Medical therapy with paracetamol was started leading to complete PDA closure. On the fourth day of life, extubation was performed and nasal CPAP support was started.

**Figure 1:**
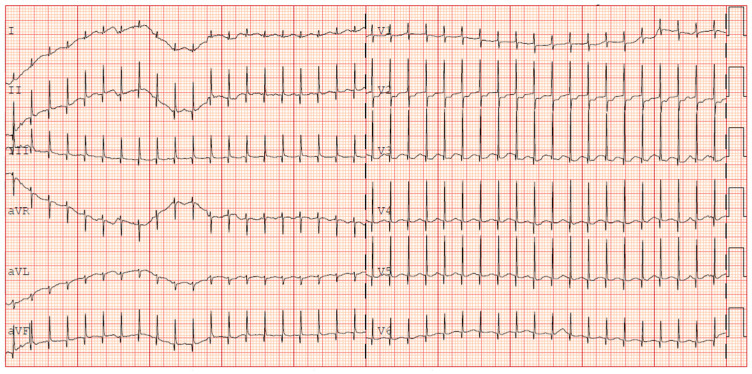
ECG occurrence of supraventricular tachycardia (+31 days of life).

The following clinical course was uneventful until the fourth week of life when multiple apnoea episodes unresponsive to caffeine and doxapram therapies required reintubation. In clinical suspicion of late onset sepsis, empiric therapy with meropenem and vancomycin was started, and blood culture was performed (negative). CSF (cerebrospinal fluid) analysis was unreliable due to hematic contamination, and culture was negative. Repeated echocardiography showed sub-acute pericardial effusion with normal biventricular function and no associated valvular abnormalities. After 48 h from the onset of the symptoms, a 12-lead ECG tracing was performed during a sudden tachycardia episode, revealing a narrow QRS complex tachycardia (HR 240 bpm) without visible P waves preceding each QRS, suggestive of junctional pattern. No delta waves were observed, excluding the presence of an accessory pathway (e.g., WPW syndrome). A transient response to vagal stimulation confirmed a supraventricular origin. PR intervals were not consistently measurable due to the atrioventricular dissociation, and retrograde P waves were not identifiable, further supporting a junctional origin (Figure 1).

Epicutaneo-caval catheter location was outside the atrium and no electrolyte imbalance was identified. A slight increase in troponin I was reported (0.8 ng/mL) in presence of markedly raised NT pro-BNP (19.000 pg/nl). Due to the recurrence of SVT episodes, therapy with propranolol was started. Complete blood tests showed an elevation of transaminases (AST 128 U/L—ALT 117 U/L). Mild persistent c-reactive protein (CRP) elevation was noted despite antibiotic therapy (0.91 mg/dl). Considering the association between hepatic and cardiac involvement, a viral multiplex PCR screening was performed on a blood sample revealing the presence of enterovirus RNA.

**Figure 2:**
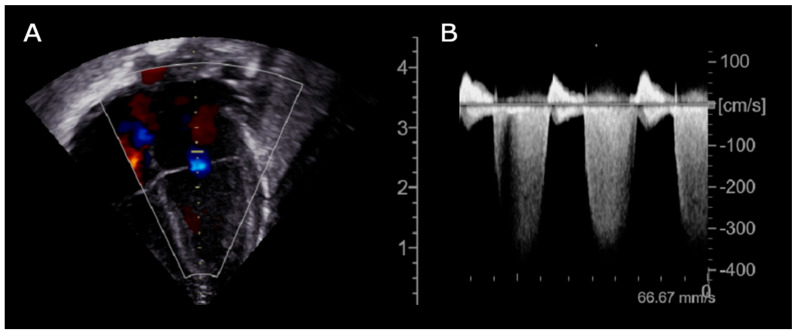
Echocardiogram (day +36), (**A**): Apical 4-chamber B-mode showing mitral regurgitation; (**B**): Continuous wave Doppler tracing of the mitral valve demonstrating a dense, holosystolic regurgitant jet with peak velocity exceeding 4 m/s. The complete systolic envelope and high signal intensity are indicative of significant mitral regurgitation with elevated left ventricular–left atrial pressure gradient.

Prompt transfontanellar ultrasound showed low-grade bilateral IVH with no additional abnormalities. Brain and cardiac MRI were considered unfeasible due to the unsafe clinical condition (increased SVT episodes frequency). Given the partial response to propranolol treatment, flecainide was added as second-line therapy. Subsequent echocardiography showed new onset of focal hypokinesia of the middle lower segment of the left ventricle, mild mitral regurgitation and enlargement of the left atrium (Figure 2).

**Figure 3:**
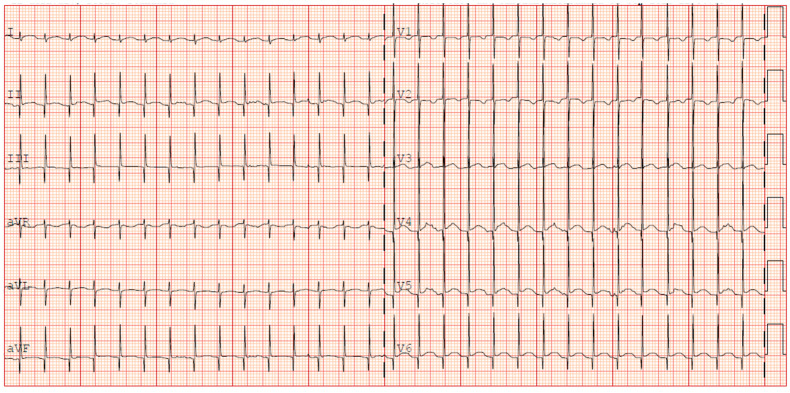
ECG persistence of junctional rhythm during propranolol and fleicanide therapy (+35 days).

Given the progressive increase in the frequency of SVT episodes, a pharmacological cardioversion trial with adenosine was administered, resulting in a transient response and a reset of the incessant atrial tachycardia. It was then decided to discontinue flecainide and initiate amiodarone therapy (with loading i.v. dose). Given no improvement in SVT control in 48 h, digoxin was also started. This triple therapy resulted in a reduction in the heart rate to values compatible with gestational age (HR 140–160 bpm); however, it did not achieve a stable sinus rhythm. During junctional rhythm (Figure 3), the ECG showed regular QRS complexes with absent or retrograde P waves and a relatively fixed RR interval. The P wave axis was inferiorly directed when visible, indicating an origin from low atrial or junctional tissue. PR intervals remained unstable or inverted. These features suggested a likely diagnosis of junctional ectopic tachycardia (JET) or ectopic atrial tachycardia (EAT), although precise classification was limited by the infant’s clinical condition and the lack of an electrophysiology study.

Enterovirus RNA on blood was still positive at 12 weeks after onset of the symptoms. Given the absence retrieval of a sinus rhythm to digoxin and amiodarone therapy, these two drugs were discontinued at approximately three months of age.

**Figure 4:**
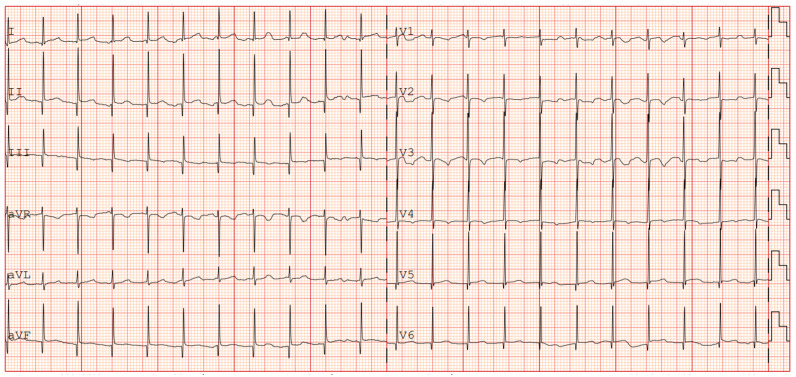
ECG showing return to atrial rhythm (during propranolol therapy).

**Figure 5:**
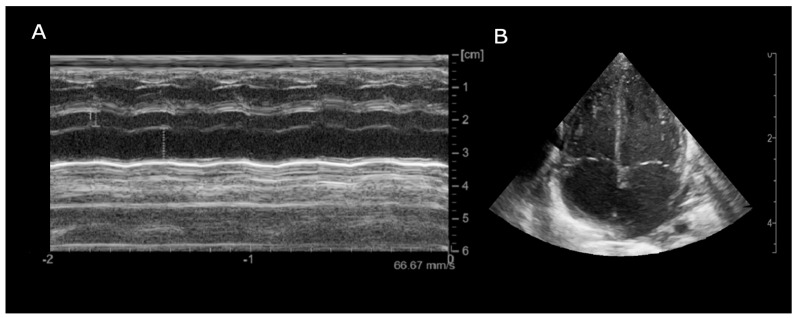
Echocardiogram (month +8), (**A**): Parasternal long-axis M-mode (LA/Ao ratio 2.18); (**B**): Apical 4-chamber B-mode showing bilateral atrial enlargement.

The patient was then discharged with stable cardiac function and propranolol therapy with the exclusive aim of controlling the heart rate. Serial electrocardiographic evaluations showed persistence of accelerated junctional rhythm until the seventh month of life, when, during a short hospitalization due to Influenza B virus, the appearance of atrial rhythm was identified (Figure 4). The last echocardiography showed normal biventricular function (EF 55–60%) with persistent bi-atrial enlargement (Figure 5).

One month after, the patient was in good clinical condition with serial ECG showing a stable heart rate with few atrial P-waves under propranolol prophylaxis.

Written informed consent was obtained from the parents of the reported patient.

## 3. Discussion

This case illustrates a rare and complex presentation of enterovirus myocarditis in an ELBW infant, characterized by persistent supraventricular arrhythmia and delayed conversion to atrial rhythm. The diagnostic workup was limited due to the infant’s instability, which precluded cardiac MRI and biopsy, and thus required reliance on clinical and virological findings.

Cardiological involvement during enterovirus infection can lead to a severe clinical picture with the need for complex treatment. Immunohistochemical analysis (immunofluorescence assays) showed that evidence of enterovirus in cardiac tissue is much more frequent than in adult cases [11]. In multiple case series with sudden infant death syndrome, enterovirus was detected from post-mortem myocardial samples. [12,13]. This data is apparently controversial when compared to different studies [14].

Receptors expressed on the cell surface of cardiomyocytes represent the target of certain types of enteroviruses (e.g., coxsackie B viruses) [15]. Enterovirus has also showed the capability to induce cardiomyocyte apoptotic pathway by an up-regulation of Bax expression, potentially causing a progression of the disease [16]. Specific enterovirus expressed proteases (2Apro and 3Cpro) appear also to be correlated with the persistence of viral infection and tropism for cardiac proteins (e.g., dystrophin) [15]. In the adult population, enteroviruses harboring a 5′ terminal genomic RNA deletion tend to become chronic and have been associated with the pathogenesis of unexplained dilated cardiomyopathies [17]. These multiple mechanisms have been postulated in the development of cardiac conduction disturbances, cardiac injury, and inflammation [15,16].

Historically, the diagnosis of myocarditis has been related to direct tissue examination using Dallas criteria [18]. Nowadays, myocarditis diagnosis is pursued with a mult-modal approach using molecular technique and functional cardiac exams (such as echocardiography and cardiac magnetic resonance) [19]. However, it is important to emphasize that the isolation of a viral agent in peripheral samples can only be regarded as an alternative to myocardial viral PCR if the latter is deemed to be unfeasible [19]. In our patient, given the ELBW and unstable clinical conditions, myocardial biopsy and cardiac MRI were considered unsafe and non-diagnostic.

Multiple case series have been reported regarding a newborn with arrhythmia due to EV infection, as summarized in Table 1. The most frequently identified arrhythmia is supraventricular tachycardia (SVT, in addition to JT), followed by premature complexes (ventricular or atrial). Occasionally, atrial flutter and ventricular tachycardia have been reported. All the patients have been diagnosed with EV infection by analyses performed on stools, CSF, or blood. No biopsies or cardiac MRI have been performed.

The ECG features observed in our patient, specifically the absence of pre-excitation, unstable PR intervals, and lack of P waves preceding QRS, are consistent with a diagnosis of JET or focal atrial tachycardia, rather than reentrant SVT. Although an electrophysiological study was not feasible, the rhythm’s resistance to vagal maneuvers and partial response to multi-agent therapy support an automatic rather than a reentrant mechanism. We interpret this finding as a post-inflammatory conduction disturbance, possibly reflecting autonomic immaturity or transient injury to the sinus node or atrial conduction pathways following enterovirus myocarditis.

Additionally, in evaluating the clinical course of our patient, the possibility of tachycardia-induced cardiomyopathy (TIC) should also be considered. TIC is a reversible form of myocardial dysfunction resulting from persistent or recurrent tachyarrhythmias. Although our patient did not develop overt heart failure or severe systolic impairment, echocardiographic findings revealed focal hypokinesia and mild left ventricular dysfunction, coinciding with a period of sustained supraventricular tachycardia. These abnormalities gradually improved following multi-agent antiarrhythmic therapy, supporting the hypothesis of early-stage TIC. Given the immature myocardial reserve in ELBW infants, even moderate rhythm disturbances may precipitate subclinical myocardial impairment. While definitive diagnosis of TIC is challenging in this setting due to limitations in diagnostic modalities (e.g., strain imaging or cardiac MRI), this case illustrates the need to consider arrhythmia-related myocardial dysfunction within the broader pathophysiological framework of enteroviral myocarditis in neonates.

Antiarrhythmic treatment was administered in a stepwise manner over time, based on clinical response. Propranolol was initially introduced following recurrent SVT episodes. Due to limited efficacy, flecainide was added but discontinued after the appearance of left ventricular hypokinesia and persistent tachyarrhythmia. Amiodarone was then started with an intravenous loading dose; however, after 48 h of limited response and continued hemodynamic instability, digoxin was cautiously introduced. This decision was based on the absence of delta waves or signs of pre-excitation, with the goal of enhancing atrioventricular conduction and achieving better rate control. Continuous telemetry and frequent ECG monitoring were performed to mitigate the risk of proarrhythmia. Ultimately, the triple therapy helped reduce the heart rate to a range more compatible with gestational age, although sinus rhythm was not restored.

It should be noted that there is no specific antiarrhythmic drug preferred in patients with myocarditis [19]. Our antiarrhythmic approach is consistent with the most recent recommendations outlined in the 2024 American Heart Association (AHA) Scientific Statement on the pharmacological management of cardiac arrhythmias in the fetal and neonatal periods [20]. According to these guidelines, adenosine remains the first-line treatment for acute termination of SVT when vagal maneuvers are ineffective. For ongoing rhythm control and prevention of recurrence, beta-blockers (such as propranolol) and digoxin are considered appropriate, especially in the absence of pre-excitation. The AHA 2024 statement also emphasizes that multi-drug regimens may be required in refractory cases, particularly when automatic tachycardias are suspected [21]. In our patient, the use of amiodarone and digoxin in combination, though approached cautiously, reflected this escalation strategy in a clinically unstable, preterm infant with persistent arrhythmia unresponsive to first- and second-line agents. Long-term therapy is recommended for approximately 6–12 months after SVT [22].

To the best of our knowledge, this is the first described case of EV-induced arrhythmia occurring in an ELBW newborn with FGR. Given the more profound immaturity of the autonomic system and the fragility of cardiovascular regulation, the treatment must be timely and shared with pediatric cardiologists. In addition, recurrent SVT has been proposed as a risk factor for necrotizing enterocolitis [23].

In EV-induced myocarditis survivors, the burden of long-term prognosis can be characterized by the development of cardiac comorbidities in up to 66% of patients (severe dysfunction of the left ventricle or mitral valve regurgitation) [24].

**Table 1:** Reported arrhythmias in enterovirus associated myocarditis in NICU.

Pt.	Type of Arrhythmia	GW	BW (g)	Onset (DOL)	Diagnosis Method (Virus)	Antiarrhythmic Treatment	Follow Up (months)	Ref.	Pt. Nationality
1	AF—SVT	36	3700	1	Stools (CoxsackieB2)	Electric Cardioversion (2J) + Digoxin until 6 months	AW (18)	[25]	USA
2	Intermittent JT	36	N/A	6	CSF (Enterovirus)	Electric Cardioversion + Amiodarone + Propranolol (→ Sotalol) + Digoxin	AW (1)	[1]	USA
3	Complete AVB, intermittent JT	30	N/A	30	Blood (Enterovirus)	Atropine + Digoxin	Recurrence AF (1) LV dilatation (3)		
4	AF	27	N/A	39	CSF (Enterovirus)	Adenosine → Digoxin + Electric Cardioversion → Amiodarone + Propanolol	Moderate bi-atrial dilation, no arrhythmia (5)		
5	PVC, ventricular bigeminy	34 + 5	2460	9	CSF, blood, stools (Enterovirus CVB5)	BB	No follow-up data reported	[26]	Italy
6	PAC, PVC, SVT	35	3035	2	CSF (CoxsackieB1)	Not reported	AW (no timing reported)	[3]	USA
7	SVT, ST depression	38	1770	38	Stools (ECHO 11)	Digoxin	No follow-up data reported	[27]	Australia
8	SVT, ST depression	34	1870	25	Stools (ECHO 11)	Digoxin	No follow-up data reported		
9	SVT	26	846	31	Stools (ECHO 11)	Digoxin	No follow-up data reported		
10	SVT, JET, EAT, VT, PAC, PVC, ST abnormalities	37 + 6	3365	7	Stools, blood (Enterovirus)	Adenosine + Cardioversion + BB	AW (8)	[28]	Republic of Korea
11	SVT	38	3400	11	Blood (Enterovirus)	Adenosine + Amiodarone+ Propranolol	No follow-up data reported	[29]	Croatia
12	JET	37	2200	11	Blood (ECHO 6)	Adenosine + Electric cardioversion + Digoxin + Amiodarone + Carvedilol	AW (3)	[30]	Germany
13	AVR, PVC	Term	3400	1	Blood, Antibodies (Coxsackie B4)	Antiarrhythmic (not specified)	AW (36)	[31]	Japan
14	SVT	27 + 6	760	29	Blood	Adenosine, Propranolol, Flecainide (→ Digoxin), Amiodarone	AW (7)	Our case	Italy

AF: atrial flutter; AVB: atrio-ventricular block; AVR: accelerated ventricular rhythm; AW: alive and well; BB: beta-blockers; BW: birth weight, CSF: cerebrospinal fluid; DOL: days of life; EAT: ectopic atrial tachycardia; EF: ejection fraction; JET: junctional ectopic tachycardia; JT: junctional tachycardia; PAC: premature atrial complexes; PVC: premature ventricular complexes; VT: ventricular tachycardia.

The persistence of bi-atrial enlargement on the final echocardiogram, despite normalization of systolic function, may reflect several underlying mechanisms. Chronic tachyarrhythmia during a critical developmental period could have contributed to structural atrial remodeling. In addition, prior elevated filling pressures or subtle residual diastolic dysfunction, common in the aftermath of tachycardia-induced cardiomyopathy, may have played a role. While left ventricular systolic function was within normal limits (EF 55–60%), comprehensive diastolic assessment was limited. Pulsed-wave Doppler evaluation of mitral inflow did not reveal restrictive or pseudonormal patterns; however, tissue Doppler imaging could not be reliably performed due to the infant’s low body weight and technical limitations. Therefore, we cannot entirely exclude the presence of subclinical diastolic dysfunction, and close cardiological follow-up has been planned given the anatomical findings and history of arrhythmia.

In this context, serial echocardiography and ECG evaluations should be established as part of the longitudinal follow-up of these patients. Educating the caregivers in recognizing arrhythmia symptoms should also be sought.

This report adds to the literature by documenting the first case of EV-induced myocarditis with prolonged arrhythmia in this group of patients. It emphasizes the importance of early virological evaluation, multi-disciplinary management, and sustained follow-up, given the potential for delayed rhythm normalization and persistent cardiac abnormalities. Therapeutically, the case underscores the need for individualized, stepwise, and prolonged antiarrhythmic strategies in preterm patients.

## 4. Conclusions

Enterovirus infection in the neonatal period is a condition that can be life-threatening due to the presence of a possible immature conduction system, which may predispose individuals to an increased risk of developing arrhythmias. In the case of patients with late-onset sepsis that is unresponsive to therapy, virological work-up must also be considered. The management of these patients must be shared between neonatologists and pediatric cardiologists. The propensity for cardiological complications necessitates ongoing instrumental and functional evaluations during follow-up. In conclusion, investigating the molecular mechanisms underlying the cardiac tropism of enteroviruses is crucial for identifying new diagnostic and therapeutic approaches.

## Data Availability

The datasets generated during and/or analyzed during the current study are not publicly available but are available from the corresponding author on reasonable request.

## Abbreviations

The following abbreviations are used in this manuscript:

NICU	Neonatal Intensive Care Unit
DOL	Days of Life
VT	Ventricular Tachycardia
SVT	Supraventricular Tachycardia
ECG	Electrocardiogram
ECMO	Extracorporeal Membrane Oxygenation
NAs	Neonatal Arrhythmias
AV	Atrioventricular
VF	Ventricular Fibrillation
ELBW	Extremely Low Birth Weight
LBW	Low Birth Weight
AVRT	Atrioventricular Reentrant Tachycardia
WPW	Wolff–Parkinson–White syndrome
AVNRT	Atrioventricular Nodal Reentrant Tachycardia
PJRT	Permanent Junctional Reciprocating Tachycardia
JET	Junctional Ectopic Tachycardia
GW	Gestational Weeks
FGR	Fetal Growth Restriction
FiO2	Fraction of Inspired Oxygen
BID	Bis in Die
PDA	Patent Ductus Arteriosus
CPAP	Continuous Positive Airway Pressure
LOS	Late Onset Sepsis
CSF	Cerebrospinal Fluid
NT-proBNP	N-terminal pro B-type Natriuretic Peptide
AST	Aspartate Aminotransferase
ALT	Alanine Aminotransferase
CRP	C-Reactive Protein
MRI	Magnetic Resonance Imaging
IVH	Intraventricular Hemorrhage
EAT	Ectopic Atrial Tachycardia
EF	Ejection Fraction
